# Development and psychometric testing of a questionnaire to assess Nurse’s perception of risks during enteral nutrition

**DOI:** 10.1186/s12912-020-00520-z

**Published:** 2021-01-05

**Authors:** Ping Feng, Hui-Li Yang, Lan Xu, Omorogieva Ojo, Xiao-Yan Lu, Hai-Ying Zhang, Xiao-Hua Wang

**Affiliations:** 1 Department of Gastroenterology, The First Affiliated Hospital of Soochow University, Suzhou, 215006 China; 2 School of Nursing, Medical College of Soochow University, Suzhou, 215006 China; 3 Nursing department, The First Affiliated Hospital of Soochow University, Suzhou, 215006 China; 4 Department of Adult Nursing and Paramedic Science, University of Greenwich, London, SE9 2UG UK; 5 Department of Intensive Care Unit, The First Affiliated Hospital of Soochow University, Suzhou, 215006 China; 6 Department of Neurosurgery, The First Affiliated Hospital of Soochow University, Suzhou, 215006 China; 7 Department of Cardiology, The First Affiliated Hospital of Soochow University, Suzhou, 215006 China

**Keywords:** Enteral nutrition, Nurses, Risk perception, Instrument development

## Abstract

**Background:**

Enteral nutrition (EN) therapy is widely used in clinical practice to provide artificial nutrition to patients, while the incidence of adverse events are relatively highly. In the clinical setting, the occurrence of adverse events is associated with the nurse’s risk perception. Thus, using tool to evaluate nurse’s risk perception of enteral nutrition is necessary.

**Methods:**

The draft questionnaire with 37-items was formed by comprehensive literature reviews and semi-structured in-depth interviews with 11 nurses. Two iterations of expert consultations were used to evaluate the content validity, and 4 items were deleted in this phrase. A 33-items questionnaire was used to survey 352 nurses from five tertiary hospitals in China from May to July 2019 with convenience sampling. Content validity, construct validity and known-groups validity were evaluated by content validity index (CVI), exploratory factor analysis, and the comparisons of the different EN risk perception levels of nurses at different working departments and different educational backgrounds, respectively. Reliability was tested by internal consistency, test-retest reliability, and split-half reliability.

**Results:**

After the exploratory factor analysis, four items were excluded. Finally, the newly developed questionnaire included 29 items explaining 71.356% of the total variance. It consisted of three factors: Risks of operation (15 items); Risks of EN-related adverse events (11 items), and Risks of EN solution selection (3 items). The CVI of the questionnaire was 0.95 and the CVI of items ranged from 0.875–1.0. The results of known-groups validity showed that the nurses with different educational backgrounds had a statistically significant difference of EN risk perception (*z* = − 3.024, *p* = 0.002), whereas there was not significantly different between EN risk perception of nurses working in different departments (*z* = − 1.644, *p* = 0.100). The Cronbach’s α, test-retest reliability, and split-half reliability of the questionnaire were 0.967, 0.818, and 0.815, respectively.

**Conclusions:**

The newly developed questionnaire for assessing nurse’s EN risk perception showed good reliability and validity. It can be used as a tool for nursing managers to assess Chinese nurses’ EN risk perception ability, so as to help to reduce the occurrence of adverse events during EN implementation.

**Supplementary Information:**

The online version contains supplementary material available at 10.1186/s12912-020-00520-z.

## Background

Enteral nutrition (EN) is an effective method of providing nutritional support to patients with functional gastrointestinal tract who are unable to meet their nutritional requirements orally [1, 2]. Due to its convenient use characteristics and therapeutic advantages, it has been widely used in clinical practice. Compared with parenteral nutrition, timely and suitable EN support can maintain the function of the intestinal barrier, has a lower risk of infection and better clinical outcomes [3–5].

However, adverse events usually occur throughout the whole process of EN therapy. The adverse events can be usually divided into four major categories: (1) mechanical, e.g., tube blockage or removal; (2) gastrointestinal e.g., nausea, diarrhea; (3) infectious e.g., aspiration pneumonia, tube site infection; (4) metabolic, e.g., refeeding syndrome [6]. The frequency of gastric tube removal has been reported to be 10.2 removals for 1000 device days in intensive care unit (ICU), the highest incidence among all devices-removed [7]. Diarrhea is the most commonly reported gastrointestinal side effect, which is estimated to occur in 15–18% of critical care patients who receive EN therapy [8, 9]. Aspiration pneumonia is a potentially life-threatening complication, it can occur in critical care patients and patients with neurological problems. The incidence of aspiration has been reported to reach 20% of enteral nutrition patients [8]. Hypophosphatemia is one of the most common refeeding syndrome, which was found that it occurs in almost half of ICU patients who were started on enteral feeding [4, 10].

In the clinical setting, the occurrence of adverse events is associated with the nurse’s risk perception [11]. The risk perception refers to the individual’s perception and understanding of various objective risks in the outside world [12]. Behavioral theories suggest that a high risk perception encourages individuals to adopt actions to reduce the risk [13]. Sellick et al. found that the incidence of needle stab injury was significantly lower after training nurses on nursing risk perception [14]. Wen et al. concluded that clinical nurse’s score of risk perception of adverse events is negatively correlated with patients’ safety, indicating that the stronger nurse’s ability to perceive risk, the safer patient was [11]. Oyapero et al. found that student nurses with a higher risk perception were more positive likely in the behavior of hand hygiene (OR = 1.54; 95% CI: 1.03–2.51) [15].

Based on the above, we can infer that the nurse’s risk perception is associated with the incidence of adverse events of EN. But until now, few studies about this aspect were reported. Pre-assessment of risk perception may reduce the occurrence of negative events. Meehan et al. reported that a nurse-initiated, perioperative pressure injury risk assessment measure resulted in a 60% reduction in pressure injuries [16]. Using tools with good reliability and validity to assess nurses’ risk perception of EN may help to identify nurses with low level of EN risk perception, then strengthen training EN-related knowledge and skills, thereby reducing incidence of adverse events. However, there are no relevant valid tools to be found for assessing it so far. In this case, the purpose of this study was to develop a questionnaire for assessing nurses’ risk perception of EN and subsequently tested its psychometrical characteristics.

## Methods

### Aims and questions

The purpose of this study was to develop and psychometrically test the questionnaire, a new instrument to measure risk perception of EN among nurses, for addressing three questions: (1) what are the psychometric properties of the questionnaire; (2) what are the factors of the questionnaire; (3) if the questionnaire can reliably and effectively measure the EN risk perception among Chinese nurses.

## Methodology

This is a methodological questionnaire development study, which includes three phases: (1) formation of the questionnaire; (2) preliminary item evaluation; (3) questionnaire refinement and psychometric evaluation.

### Formation of the questionnaire

The item pool was generated based on reviewing the literature, theories, and relevant questionnaires about risk perception among nurses, and semi-structured interviews with 11 nurses. We noticed that EN therapy includes initiation, monitoring, maintenance and termination, of which, monitoring and maintenance were the most important stages, in which most adverse events happened in these two stages after reviewing articles about the EN-related risks. Thus, two factors, namely, Risks of operation and Risks of EN-related adverse events were considered as the prior factors of the questionnaire. Additionally, the theory of risk perception was referenced to ensure that the item pool was theoretically coherent. For the reasonable expression and construction of items, the instrument developed by Zhang et al. [17] for assessing nurses’ occupational risk perception and the tool developed by Mao et al. for assessing nurses’ risk perception of the nursing adverse events were referenced to form the initial items of this questionnaire [18].

The semi-structured in-depth interviews were conducted in a meeting room in the hospital. When the information is saturated, new interviews would not be conducted. Finally, 11 nurses with more than one year of work experience and working in different departments including the internal medicine department, surgical department, emergency department and ICU participated in the interview. The main questions included the following: a) What kind of risks do you think exist during the process of EN? b) Please rank these risks according to the severity of the consequence of the risk to the patients. Other information including the interviewee’s expansion of the question, facial expressions and gestures were also recorded. Data were analyzed using phenomenology analysis. Based on the above, the other three factors were formed: Risks of EN-solution selection, Risks of knowledge deficit, and Risks of patients’ own causes.

According to the measurement method of risk perception proposed by Culmingham [19], the ability of risk perception was measured by multiplying the scores of the probability of risk occurrence and the severity of the consequence of the risk, these two parts were directly investigated by items. The probability of risk occurrence was rated on a six-point Likert questionnaire (1 = extremely unlikely to 6 = extremely likely), the corresponding score was 1–6 points. The severity of the consequence of risk was rated on a five-point Likert questionnaire (1 = not serious at all to 5 = very serious), the corresponding score was 1–5 points. The score of each item was calculated by multiplying the scores of these two parts. The total score of the whole questionnaire was calculated according to the formula of (actual score / highest score) × 100. The higher the nurse’s risk perception score, the higher the level of risk perception.

After integrating the obtained information, a draft questionnaire with 37-items was formed: 12 items for the factor “Risks of EN-related adverse events”, 17 items for the factor “Risks of the operation”, 2 items for the factor “Risks of EN solution selection”, 4 items for the factor “Risks of knowledge deficit” and 2 items for the factor “Risks of patients’ own causes”.

### Preliminary item evaluation

To validate the content, a panel of experts, including seven ICU nurses, four clinicians, two nursing educational experts, a physical therapist, a psychologist, and an expert who is proficient in questionnaire development, was asked to evaluate the necessity and integrity of each item. Each expert was asked to rate each item for relevance on a five-point Likert questionnaire: 1 = highly irrelevant, 2 = not relevant, 3 = not decided, 4 = relevant, 5 = highly relevant. Additionally, experts were asked to give advice on whether items were appropriately phrased and if there were additional factors or items need to be added.

After initial round of expert consultation, the following changes were made: one item “without evaluating the speed of infusion pump during the implement of EN” was deleted because its statement was not in conformity with the gastrointestinal peristaltic demand; seven items were amended due to improper expression. In the secondary round of expert consultation, three items were removed. The item “without selecting EN solution according to the patient’s condition” was deleted because its meaning was similar to the item “without choosing the appropriate EN solution based on the changes in disease”, another two items “difficulty may exist in extubating after EN” and “without cleaning the syringe immediately after EN” were deleted because the experts rated them on a low score of relevance. No item was added during the expert consultation. So the questionnaire containing 33 items was formed after two iterations of content validation.

### Questionnaire refinement and psychometric evaluation

The instrument was constructed in Chinese because it was developed for assessing the risk perception of EN among Chinese nurses. For face validity, the initial questionnaire was conducted with 10 nurses who once administered EN to patients and have more than 1 year of work experience. According to their opinions, the intelligibility of each item was good, so no item was modified.

### Participants and setting

Nurses from five tertiary general hospitals in Jiangsu, Zhejiang, and Shanghai provinces in China were enrolled. Convenience sampling was used in this study. Registered nurses who have worked for more than one year and had experience of EN administering were invited to the study. Registered nurses who did not provide direct clinical care and training nurses were excluded.

### Sampling

It has been suggested that, when developing a new questionnaire, the sample size should be 5–10 times greater than the total number of items in the questionnaire [20]. In consideration of the pretested questionnaire had 33 items and a 20% drop-out rate, the sample size should be 198–396. Ten percent of total samples are selected to refill the questionnaire at a time when 10 days later after first time investigation.

### Ethical considerations

The study was approved by the ethics committee of the first affiliated hospital of Soochow University (ethnic number: 2019008). The participants were informed of the purpose and procedures of the study and signed the written informed consent.

### Data collection

Data were collected by the researcher or uniformly trained investigators via face-to-face interviews with the nurses. Participation was voluntary and anonymous. After signed the informed consent, nurses completed the general information questionnaire including socio-demographic variables (age, sex, educational background, working department, working years, professional title) and the surveyed version of the questionnaire. It took about 15 to 20 min for the respondent to complete a questionnaire. The data collection lasted from May to August 2019.

### Determination of validity and reliability

The validity was determined by content validity, construct validity and known-groups validity which were evaluated by content validity index (CVI), exploratory factor analysis, and the comparisons of the different EN risk perception levels of nurses at different working departments and different educational backgrounds, respectively. The study of Chen et al. found that nurses’ occupational risk perception showed significant difference among nurses with different educational backgrounds and working departments [21]. Therefore, assuming that the newly developed questionnaire has good know-groups validity, it could reflect different levels of EN risk perception of nurses in different educational backgrounds and different work departments.

The reliability was tested by internal consistency, test-retest reliability, and split-half reliability. Internal consistency was assessed using Cronbach’s α coefficient to determine the extent that all items in a test measure the same concept [22]. The intra-class correlation coefficient (ICC) was calculated to measure test-retest reliability and split-half reliability was calculated with the Spearman Brown correlation coefficient.

### Data analysis

Statistics were performed using the SPSS 20.0 software. Demographic characteristics were analyzed using descriptive statistics. *P* ≤ 0.05 was considered significant. The normality of the data was tested before using Pearson correlation test to evaluate the item-total correlations and item-total correlation > 0.4 was considered acceptable [23]. Because the items of the questionnaire were measured using Likert score, producing sequential categorical variables, the polychoric correlation was used to generate the correlation matrix. In the evaluation of construct validity, exploratory factor analysis was performed. Before the factorial analysis, the difference between the correlation matrix and the unit matrix was evaluated by Bartlett’s test of sphericity, and the measure of sampling adequacy was evaluated by the Kaiser-Meyer-Olkin (KMO). The KMO test value > 0.6 indicated that factor analysis could be performed [24]. Principal component analysis with varimax rotation was done to extract the common factor. The extraction criterions of a factor were following: (1) an eigenvalue of at least 1.00; (2) a factor variance of at least 5%; (3) a total variance of least 60%. Item was distributed to a factor if factor loading was > 0.40 [25] and items have adequate factor loading value between two different factors, the difference would be at least 0.20 [26]. The factor was deleted if it did not have at least three items [27, 28]. Known-groups validity was evaluated by the Mann-Whitney test to determine whether the questionnaire was able to discriminate the different levels of EN risk perception of nurses in different educational backgrounds and working departments.

The value of Cronbach’s α ≥ 0.7 indicates acceptable reliability [29]. If the Cronbach’s α coefficient increase after deleting an item, the attribution of this item was considered to be different from the other items, thus this item was removed. The values of ICC > 0.9, 0.75–0.90, 0.5–0.75 and < 0.5 indicate excellent, good, moderate and poor reliability, respectively [30]. Split-half reliability was calculated with the Spearman Brown correlation coefficient.

## Results

### Study participants

Of the 360 participants surveyed, 352 (97.8%) completed the questionnaire. Of these, 94.6% were female; 71.3% were under the age of 30 years; 80.4% had a bachelor’s degree or higher; 78.4% had a professional title with senior nurses or below. The socio-demographic characteristics were presented in Table 1. Thirty-four participants were selected from the sample to refill the questionnaire.

**Table 1:** Socio-demographic characteristics of participants (*n* = 352)

Demographic features	*n* (%)
Gender	
Female	333 (94.6)
Age, years	
≤ 30	251 (71.3)
31–40	79 (22.4)
41–50	20 (5.7)
51–60	2 (0.6)
Educational background	
Junior college	62 (17.6)
Bachelor degree	283 (80.4)
Graduate degree or above	7 (2.0)
Working duration, years	
≤ 2	92 (26.1)
3–5	102 (29.0)
6–10	86 (24.4)
11–20	51 (14.5)
≥ 21	21 (6.0)
Professional title	
Senior nurses or below	276 (78.4)
Chief nurse	70 (19.9)
Others	6 (1.7)
Department	
ICU	126 (35.8)
Internal medicine	77 (21.9)
Surgical	139 (39.5)
Emergency	10 (2.8)

### Validity

#### Content validity

The scale content validity index (S-CVI) was 0.95 and the item content validity index (I-CVI) ranged from 0.875–1.0.

#### Preliminary factor analysis

The KMO value was 0.959 (χ^2^ = 10,167.543, *p* < 0.001) and Bartlett’s test of sphericity (χ^2^ = 11,671.034, *p* < 0.001) was significant, indicating that factor analysis can be performed. The first round of exploratory factor analysis was conducted with a 33-items questionnaire. Four factors were extracted with an eigenvalue greater than 1, explaining 72.696% of the variance. The factor loading of all items was above 0.40. Total of four items were removed from the questionnaire according to the exclusion criterion. Of which, two items named “Doctor choose EN solution by objective judgment” and item “Medical staff doesn’t identify adverse events of EN timely” showed the cross-loading. Another two items “patient pulls out the tube accidentally” and “patients’ family prepares food for EN by themself” formed a independent factor which did conform to the pre-set principle that a factor should contain at least three items. As a result, the remaining items of questionnaire were 29.

#### Final factor analysis

**Fig. 1:**
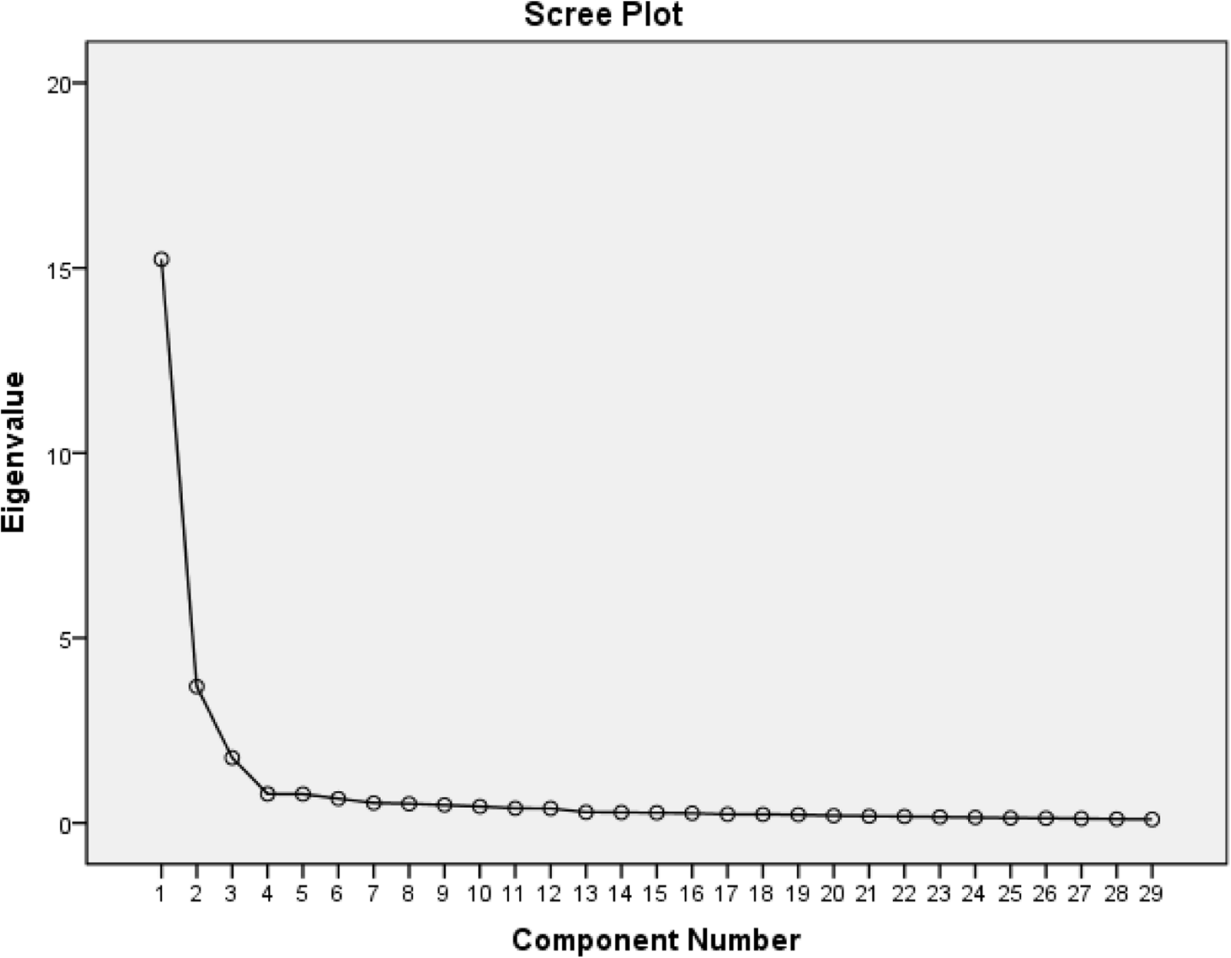
scree plot

Three factors extracted accounted 71.356% of the total variance. The factor loading of items were all acceptable. The results of the final exploratory factor analysis were presented in Table 2. To evaluate the accuracy of the number of factors, the scree plot was used and it confirmed the three-factor structure (Fig. 1).

**Table 2:** Item factor loading, item-total correlations, and the variances explained

Items	Factors			Item-total correlation
	1	2	3	
Without a flushing tube correctly after administering EN.	**0.858**	0.254	0.128	0.687
Without confirming gastric retention before administering EN.	**0.833**	0.248	0.236	0.677
Without confirming the location of the tube before administering EN.	**0.817**	0.219	0.162	0.635
The pump speed of EN solution was not personalized.	**0.811**	0.221	0.274	0.716
Without elevating bed head at least 30° during EN	**0.796**	0.205	0.124	0.653
Insufficient tube placement.	**0.781**	0.328	0.189	0.701
The complications of EN were not dealt with timely.	**0.778**	0.233	0.299	0.693
The person who placed the tube was not realized that the tube was misplaced.	**0.771**	0.288	0.201	0.682
The tube was lack of identification.	**0.767**	0.305	0.124	0.671
Without using a pump to administer EN.	**0.766**	0.147	0.165	0.578
The tube was misconnected.	**0.739**	0.294	0.063	0.616
The medical staff was lack of knowledge of EN.	**0.733**	0.294	0.288	0.684
The temperature of EN solution was inappropriate.	**0.718**	0.383	0.038	0.675
Medical staff did not update the knowledge of EN timely.	**0.713**	0.312	0.368	0.703
Screening and evaluation were not performed as required during EN administering.	**0.710**	0.412	0.064	0.683
Constipation may occur during EN administering.	0.222	**0.831**	−0.054	0.585
Metabolic complications may occur during EN administering.	0.285	**0.817**	0.060	0.567
Abdominal distension may occur during EN administering.	0.234	**0.799**	0.048	0.528
Abdominal cramps may occur during EN administration.	0.291	**0.794**	0.098	0.591
Diarrhea may occur during EN administering.	0.167	**0.778**	0.100	0.508
Long-term compression of the tube causes local skin/mucosal damage.	0.203	**0.773**	0.132	0.549
Infection may occur during EN administering.	0.341	**0.728**	0.100	0.608
Aspiration may occur during EN administering.	0.247	**0.673**	0.673	0.550
Long-term use of enteral tube feeding can lead to degeneration of gastric function.	0.271	**0.709**	0.101	0.585
Tube occlusion may occur during EN administering.	0.144	**0.748**	0.153	0.481
Tube displacement may occur during EN administering.	0.353	**0.736**	0.074	0.563
The total energy intake of EN was not up to patients’ demand.	0.278	0.080	**0.891**	0.485
Without choosing the appropriate EN solution based on the changes in disease.	0.324	0.090	**0.876**	0.520
The intake of EN solution was excessive.	0.323	0.081	**0.876**	0.522
**Eigenvalue**	15.238	3.688	1.768	
**Explained variance rate (%)**	52.544	12.716	6.096	

Factor 1: Risks of operation; factor 2: Risks of EN-related adverse events; factor 3: Risks of EN solution selection. *EN* enteral nutrition

The first factor named “Risks of operation” had an eigenvalue of 15.238 and accounted for 52.544% of the total variance. It consisted of 15 items with factor loading ranging from 0.710–0.858. The second factor named “Risks of EN-related adverse events” had an eigenvalue of 3.688 and measured 12.716% of the total variance. It included 11 items and the range of factor loading was 0.673–0.831. The last factor named “Risks of EN solution selection” had an eigenvalue of 1.768 and accounted for 6.096% of the total variance. It consisted of 3 items with factor loading ranging from 0.876–0.891.

The results of known-groups validity showed that the nurses with different educational backgrounds had a significant difference of EN risk perception (*z* = − 3.024, *p* = 0.002), whereas there was not significantly different between the levels of EN risk perception of nurses working at different departments (*z* = − 1.644, *p* = 0.100). (Table 3).

**Table 3:** Known-groups validity of the scale^−^(x ± s)

Dimensions	Education		Mann Whitney-U test		Departments		Mann Whitney-U test	
	Junior college (*n* = 62)	Bachelor degree and above (*n* = 290)	z	*p*	ICU and surgical (*n* = 265)	Internal medicine and Emergency (*n* = 87)	z	*p*
Factor 1	49.83 ± 16.89	53.84 ± 15.49	−2.170	0.030*	52.39 ± 15.08	55.39 ± 17.71	−1.088	0.276
Factor 2	35.58 ± 19.12	41.54 ± 17.23	−2.925	0.003**	39.03 ± 16.82	44.93 ± 19.57	−2.167	0.030*
Factor 3	35.56 ± 17.31	42.57 ± 16.82	−3.391	0.001**	40.05 ± 16.43	45.26 ± 18.51	−2.006	0.045*
Total scale	42.95 ± 16.57	48.01 ± 14.71	−3.024	0.002**	46.05 ± 14.29	50.37 ± 17.20	−1.644	0.100

** means *p*<0.01; * means *p*<0.05. Factor 1: Risks of operation; factor 2: Risks of EN-related adverse events; factor 3: Risks of EN solution selection;^−^x ± s:mean ± SD. *EN* enteral nutrition

#### Reliability

Item-total correlations were calculated, and the results showed that all items had acceptable corrected item-total correlation, which ranged from 0.481–0.716. The Cronbach’s α value did not rise when an item was deleted. The overall Cronbach’s α of the questionnaire was 0.967, for the factors ranging from 0.939–0.970. The split-half reliability of the total questionnaire was 0.818, for the factors ranging from 0.910–0.941. The value of test-retest reliability of the total questionnaire was 0.815, for the factor ranging from 0.688–0.885. The results were shown in Table 4.

**Table 4:** Reliability of the scale

Dimensions	Cronbach’s α coefficient	Split-half coefficient	Test-retest coefficient
Factor 1	0.946	0.910	0.885
Factor 2	0.970	0.934	0.688
Factor 3	0.939	0.941	0.768
Total scale	0.967	0.818	0.815

Factor 1: Risks of operation; factor 2: Risks of EN-related adverse events; factor 3: Risks of EN solution selection. *EN* enteral nutrition

## Discussion

This study developed a questionnaire containing 29 items for assessing nurse’ risk perception of EN. The items in the questionnaire measured three factors: Risks of operation; Risks of EN-related adverse events; and Risks of EN solution selection. The results of various criteria and statistical methods showed that the newly developed questionnaire was reliable and valid for assessing nurses’ risk perception of EN.

The construct validity of the questionnaire was evaluated by exploratory factor analysis. The load value obtained in factor analysis is the critical value that determines whether an item belongs to a specific sub-factor. Usually, items with factor loading < 0.40 should be removed from the questionnaire [25]. The factor loading of all items on this questionnaire was greater than 0.40, indicating a great structure of the questionnaire. Items loaded on two different factors with a difference of value under 0.20 which stated that the items were overlapping. Thus, two items of the questionnaire were deleted. Also, a factor should contain at least three items, otherwise, the factor should be removed from the questionnaire [27, 28]. The results of exploratory factor analysis showed that two items made up a factor, thus, these two items were deleted. Finally, based on the results of the final exploratory factor analysis and scree plot, 29 items distributing three factors were extracted and explained 71.356% of the total variance.

Known-groups validity in this study refers to the questionnaire had the ability to distinguish the levels of EN risk perception among nurses with different educational backgrounds and working departments. We found that the higher the educational level the nurse had, the higher levels of EN risk perception, which was similar to the study result of Smith et al. The study found that a positive correlation between nurses’ influenza knowledge and risk perception [31]. The knowledge theory of risk perception thought that knowledge precedes perception of risk. One cannot perceive a risk of what they do not know; hence, if knowledge level is low, risk perception will be low [32]. As for screening nurses’ different levels of EN risk perception at different working departments, we combined the data of nurses working at ICU and surgical wards as a group, and the data of nurses working at internal medicine and emergency as another group, and then compared them, since nurses working in ICU and surgical often implement EN to patients, while internal medicine and emergency nurses are less exposed to EN. In addition, Chen et.al found that nurses in the ICU had higher level of occupational risk perception [21]. But in this study, the results showed that there was no statistical difference of EN risk perception among nurses working in different departments. The reasons might be that the nurse’s working department is often not fixed but rotated. Although he/she was in internal medicine at the time of the investigation, he/she might once have an experience working at ICU or surgical ward; or he/she had once implemented EN to patients, so he/she was familiar to EN. Trevino et al. found that familiarity had the potential to either increase or decrease the perception of risk to patients; however, uncertainty universally increased the perception of risk to patients [33].

Cronbach’s α coefficient was calculated to determine the internal consistency of the questionnaire and the value of the total questionnaire above 0.70 was acceptable [29]. The Cronbach’s α of the newly-developed questionnaire (0.967) and its factors (ranging from 0.946–0.970) were very high. The split-half reliability manifests that a questionnaire has adequate reliability if the values are 0.70 during the questionnaire development and adaption processes. The split-half reliability of this developed questionnaire (0.818) and its factors (ranging from 0.910–0.941) were good. Additionally, a test-retest was performed to measure the invariance of the questionnaire by time. Results of the test-retest reliability showed that except the factor “Risks of EN-related adverse events (0.688)” showed moderate test-retest reliability, the other two factors and the questionnaire had good test-retest reliability.

The study had several potential limitations. Firstly, the results were mainly based on woman nurses. It is limited to assess the ability of risk perception of man nurses in EN nursing. Secondly, the study was conducted using convenience sample rather than probabilistic sample, therefore the findings may not be generalized to nurses working in different cultures. Further study is needed to explore this problem. Thirdly, since there are no relevant questionnaires assessing risk perception of EN, we were unable to test the criterion-related validity of the questionnaire. Fourthly, we use varimax rotation to extract the common factor, which may have little less power to reveal how strong the concepts are associated. Lastly, using factor analysis with estimating error variance may lead to fewer factors to be extracted. In addition, we did not use the GLS factor analysis to examine the stability of the third factor.

## Conclusions

The current evidence suggests that the developed questionnaire is a reliable and valid tool for assessing nurses’ risk perception of EN. It can help nursing managers to identify the level of nurses’ EN risk perception and formulate measures to reduce the occurrence of EN adverse events. Besides, since there is not a similar questionnaire in literature, the questionnaire is available for studies in this area.

The questionnaire developed for this study is provided as Additional File 1.

## Supplementary Information

**Additional file 1:** Questionnaire.

## Data Availability

The datasets used and analyzed during the current study are available from the corresponding author on reasonable request.

## Abbreviations

EN: Enteral nutrition
CVI: Content validity index
ICC: Intra-class correlation coefficient
ICU: Intensive care unit
I-CVI: Item content validity index
KMO: Kaiser-Meyer-Olkin
S-CVI: Scale content validity index

## References

[1] DeLegge MH (2018). Enteral access and associated complications. Gastroenterol Clin N Am.

[2] Lord LM (2018). Enteral access devices: types, function, care, and challenges. Nutr Clin Pract.

[3] Chow R, Bruera E, Arends J, Walsh D, Strasser F, Isenring E, Del Fabbro EG, Molassiotis A, Krishnan M, Chiu L, Chiu N, Chan S, Tang TY, Lam H, Lock M, DeAngelis C (2020). Enteral and parenteral nutrition in cancer patients, a comparison of complication rates: an updated systematic review and (cumulative) meta-analysis. Support Care Cancer.

[4] Kozeniecki M, Fritzshall R (2015). Enteral nutrition for adults in the hospital setting. Nutr Clin Pract.

[5] Yao H, He C, Deng L, Liao G (2018). Enteral versus parenteral nutrition in critically ill patients with severe pancreatitis: a meta-analysis. Eur J Clin Nutr.

[6] Blumenstein I, Shastri YM, Stein J (2014). Gastroenteric tube feeding: techniques, problems and solutions. World J Gastroenterol.

[7] Galazzi A, Adamini I, Consonni D, Roselli P, Rancati D, Ghilardi G, Greco G, Salinaro G, Laquintana D (2019). Accidental removal of devices in intensive care unit: an eight-year observational study. Intens Crit Care Nurs.

[8] Toussaint E, Van Gossum A, Ballarin A, Arvanitakis M (2015). Enteral access in adults. Clin Nutr (Edinburgh, Scotland).

[9] Ukleja A, Gilbert K, Mogensen KM, Walker R, Ward CT, Ybarra J, Holcombe B (2018). Task force on standards for nutrition support: adult hospitalized patients, the American Society for Parenteral and Enteral Nutrition standards for nutrition support: adult hospitalized patients. Nutr Clin Pract.

[10] Md Ralib A, Mat Nor MB (2018). Refeeding hypophosphataemia after enteral nutrition in a Malaysian intensive care unit: risk factors and outcome. Asia Pac J Clin Nutr.

[11] Wen X, Cai C, Wang S (2017). The influence of clinical nurses’ risk perception of adverse events and shift quality on patient safety. Chin J Pract Nurs.

[12] Slovic P, Finucane ML, Peters E, MacGregor DG (2004). Risk as analysis and risk as feelings: some thoughts about affect, reason, risk, and rationality. Risk Anal.

[13] Bandura A. Social cognitive theory of self-regulation. Organ Behav Hum Decis Process. 1991;50(2):248–87 10.1016/0749-5978(91)90022-l.

[14] Sellick JA, Jr Hazamy PA, Mylotte JM (1991). Influence of an educational program and mechanical opening needle disposal boxes on occupational needlestick injuries. Infect Control Hosp Epidemiol.

[15] Oyapero A, Oyapero O (2018). An assessment of hand hygiene perception and practices among undergraduate nursing students in Lagos state: a pilot study. J Educ Health Promot.

[16] Meehan AJ, Beinlich NR, Hammonds TL (2016). A nurse-initiated perioperative pressure injury risk assessment and prevention protocol. AORN J.

[17] Zhang X, Cao G, Xu Z, Chen Z, Zhang Y, Cao B (2016). Formation of risk perception questionnaire for nurses. Chin Nurs Res.

[18] Mao Q, Zhao B, Liu C, Zhang W (2016). Structure of adverse events risk perception scale and its reliability and validity among clinical nurses. Chin Nurs Manag.

[19] Cunningham SM. The major dimensions of perceived risk. Risk Taking Inform Handling Consumer Behav. 1967:82–108.

[20] Tavs Ancıl E (2014). Measuring Attitudes and Data Analysis with SPSS (3rded.).

[21] Chen Z, Gao H, Zhang Y (2017). Analysis of the status quo and influencing factors of nurses’ risk perception in a third grade a hospital. Nurs Res China.

[22] Jensen MP (2003). Questionnaire validation: a brief guide for readers of the research literature. Clin J Pain.

[23] Wu ML. Statistical Analysis Practices in Questionnaire Development*;* Chongqing University Press: Chongqing, China. 2010. p. 158–265.

[24] Beavers AS, Lounsbury JW, Richards JK, Schuyler WH, Skolits GJ, Esquivel SL (2013). Practical considerations for using exploratory factor analysis in educational research. Pract Assess Res Eval.

[25] Kline RB (2011). Principles and Practice of Structural Equation Modeling.

[26] Seçer, I. *Psychological test development and adaptation process*. Anı.:Ankara. 2015.

[27] Chiou HJ (2010). Quantitative research and statistics: SPSS (PASW) data analysis examples.

[28] Hwang FM (2015). Structural equation modeling.

[29] Polit DF, Beck CT (2004). Nursing research: principle and methods.

[30] Koo TK, Li MY (2016). A guideline of selecting and reporting Intraclass correlation coefficients for reliability research. J Chiropractic Med.

[31] Smith S, Sim J, Halcomb E (2016). Australian general practice nurse’s knowledge, attitudes and practices regarding influenza vaccination: a cross-sectional survey. J Clin Nurs.

[32] Wildavsky A, Drake K (1990). Theories of risk perception: who fears what and why?. Dædalus..

[33] Trevino P, Green A, Middaugh D, Heo S, Beverly C, Deshpande J (2018). Nursing perception of risk in common nursing practice situations. J Healthcare Risk Manag.

