# Supplementary material for: Development and psychometric testing of a questionnaire to assess Nurse’s perception of risks during enteral nutrition
Source: BMC Nurs. 2021 Jan 5;20:6. doi: 10.1186/s12912-020-00520-z (PMC7783971; doi:10.1186/s12912-020-00520-z)
Supplement: Supplementary file 1 — Additional file 1: Questionnaire. [file 12912_2020_520_MOESM1_ESM.doc]

Interview questions

a) What kind of risks do you think exist during the process of EN?

b) Please rank these risks according to the severity of the consequence of the risk to the patients.

Questionnaire for assessing nurse’s perception of risks during enteral nutrition

| Items | Please evaluate the likelihood of risk occurring, when you work in clinical practice. | | | | | | When the followings occurs, make an assessment of the severity. | | | | |
| --- | --- | --- | --- | --- | --- | --- | --- | --- | --- | --- | --- |
|  | Extremely unlikely | Impossible | Somewhat impossible | Somewhat possible | More likely | Extremely likely | Not serious at all | Not serious | Uncertain | Serious | Very serious |
| 1. Aspiration may occur during EN administering. |  |  |  |  |  |  |  |  |  |  |  |
| 1. Diarrhea may occur during EN administering. |  |  |  |  |  |  |  |  |  |  |  |
| 1. Long-term compression of the tube causes local skin/mucosal damage. |  |  |  |  |  |  |  |  |  |  |  |
| 1. Tube occlusion may occur during EN administering. |  |  |  |  |  |  |  |  |  |  |  |
| 1. Tube displacement may occur during EN administering. |  |  |  |  |  |  |  |  |  |  |  |
| 1. Metabolic complications may occur during EN administering. |  |  |  |  |  |  |  |  |  |  |  |
| 1. Constipation may occur during EN administering. |  |  |  |  |  |  |  |  |  |  |  |
| 1. Abdominal distension may occur during EN administering. |  |  |  |  |  |  |  |  |  |  |  |
| 1. Abdominal cramps may occur during EN administration. |  |  |  |  |  |  |  |  |  |  |  |
| 1. Infection may occur during EN administering. |  |  |  |  |  |  |  |  |  |  |  |
| 1. Long-term use of enteral tube feeding can lead to degeneration of gastric function. |  |  |  |  |  |  |  |  |  |  |  |
| 1. Screening and evaluation were not performed as required during EN administering. |  |  |  |  |  |  |  |  |  |  |  |
| 1. The temperature of EN solution was inappropriate. |  |  |  |  |  |  |  |  |  |  |  |
| 1. The tube was misconnected. |  |  |  |  |  |  |  |  |  |  |  |
| 1. The tube was lack of identification. |  |  |  |  |  |  |  |  |  |  |  |
| 1. Without a flushing tube correctly after administering EN. |  |  |  |  |  |  |  |  |  |  |  |
| 1. The person who placed the tube was not realized that the tube was misplaced. |  |  |  |  |  |  |  |  |  |  |  |
| 1. Without confirming the location of the tube before administering EN. |  |  |  |  |  |  |  |  |  |  |  |
| 1. Insufficient tube placement. |  |  |  |  |  |  |  |  |  |  |  |
| 1. Without confirming gastric retention before administering EN. |  |  |  |  |  |  |  |  |  |  |  |
| 1. Without using a pump to administer EN. |  |  |  |  |  |  |  |  |  |  |  |
| 1. Without elevating bed head at least 30° during EN |  |  |  |  |  |  |  |  |  |  |  |
| 1. The complications of EN were not dealt with timely. |  |  |  |  |  |  |  |  |  |  |  |
| 1. The pump speed of EN solution was not personalized. |  |  |  |  |  |  |  |  |  |  |  |
| 1. Without choosing the appropriate EN solution based on the changes in disease. |  |  |  |  |  |  |  |  |  |  |  |
| 1. The total energy intake of EN was not up to patients’ demand. |  |  |  |  |  |  |  |  |  |  |  |
| 1. The intake of EN solution was excessive. |  |  |  |  |  |  |  |  |  |  |  |
| 1. Medical staff did not update the knowledge of EN timely. |  |  |  |  |  |  |  |  |  |  |  |
| 1. The medical staff was lack of knowledge of EN. |  |  |  |  |  |  |  |  |  |  |  |
